# Chemical and Biological Assessment of *Angelica* Roots from Different Cultivated Regions in a Chinese Herbal Decoction Danggui Buxue Tang

**DOI:** 10.1155/2013/483286

**Published:** 2013-02-05

**Authors:** Wendy L. Zhang, Ken Y. Z. Zheng, Kevin Y. Zhu, Janis Y. X. Zhan, Cathy W. C. Bi, J. P. Chen, Tina T. X. Dong, Roy C. Y. Choi, David T. W. Lau, Karl W. K. Tsim

**Affiliations:** ^1^Division of Life Science and Center for Chinese Medicine, The Hong Kong University of Science and Technology, Clear Water Bay Road, Kowloon, Hong Kong; ^2^Department of Biology, Hanshan Normal University, Chaozhou, Guangdong 521041, China

## Abstract

Roots of *Angelica sinensis* (Danggui) have been used in promoting blood circulation as herbal medicine for over 2000 years in China. Another species of *Angelica* roots called *A. gigas* is being used in Korea. To reveal the efficiency of different *Angelica* roots, the chemical and biological properties of *Angelica* roots from different cultivated regions were compared. Roots of *A. sinensis* contained higher levels of ferulic acid, Z-ligustilide, and senkyunolide A, while high amounts of butylphthalide and Z-butylenephthalide were found in *A. gigas* roots. The extracts deriving from *A. gigas* roots showed better effects in osteogenic and estrogenic properties than that of *A. sinensis* from China. However, this difference was markedly reduced when the *Angelica* roots were being prepared in a Chinese herbal decoction together with Astragali Radix as Danggui Buxue Tang. In contrast, the herbal decoction prepared from *A. sinensis* roots showed better responses in cell cultures. In addition, the extracts of *A. gigas* roots showed strong cell toxicity both as single herb and as Danggui Buxue Tang. This result revealed the distinct properties of *Angelica* roots from China and Korea suggesting the specific usage of herb in preparing a unique herbal decoction.

## 1. Introduction 

Angelica Sinensis Radix (ASR, the roots of *Angelica sinensis*; Danggui) is one of the commonly used traditional Chinese medicines (TCM), which has been used for more than 2000 years in China. The usage of *Angelica* roots was first described in *Shen Nong Bencao Jing* (200–300 A.D., Han Dynasty in China), and the recorded functions were to replenish and invigorate blood, to stop pain and to moisten the intestines. Nowadays, it is used to promote blood circulation for the treatment of menstrual disorders [1–3], to modulate the immune system [4], as well as being a laxative for chronic constipation of the aged and debilitated [5, 6]. Chinese Pharmacopoeia (2010) recorded that *Angelica* root in China is derived from roots of *Angelica sinensis* (Oliv.) Diels (family Umbellaceae) [7]. However, *Angelica gigas* Nakai is mainly found in Korea, which was also used as *Angelica* roots in Southeast Asia [8–10]. Previous study suggested that these two commonly used *Angelica *roots showed variation in their chemical compositions, pharmacological properties, and clinical efficacies [8]; however, chemical and biological comparisons between these two *Angelica* roots have not been done. These problems compromise the values of TCM or even jeopardize the safety of consumers. Here, the amounts of ferulic acid, Z-ligustilide, butylphthalide, Z-butylenephthalide, and senkyunolide A were determined in *Angelica* roots deriving from different regions. In parallel, the biological properties of these herbal extracts in stimulating osteogenic and estrogenic effects were determined in cell culture models.

*Angelica* root was prescribed in many commonly used herbal formulae. Among these formulae, Danggui Buxue Tang (DBT) is one of the most famous and simplest one, which contains two herbs: Astragali Radix (AR, roots of *Astragalus membranaceus *var*. mongholicus*) and ASR. DBT was commonly used as a health food supplement for women aliments for more than 800 years in China. Women with menopausal symptoms were prescribed to drink DBT daily to enrich their “Qi” and nourish their “Blood” [11]. Indeed, recent pharmacological results indicated that DBT had the ability to promote hematopoietic function, to stimulate cardiovascular circulation and immune response, to increase insulin sensitivity, to prevent osteoporosis, and to act as antioxidants [12–16]. In order to reveal the role of *Angelica* root in a herbal decoction, here, we compared osteogenic, estrogenic, and hematopoietic effects of DBT prepared from different *Angelica* roots.

## 2. Materials and Methods

### 2.1. Plant Materials, Chemicals, and Reagents

Fresh plants were obtained from China: *A. sinensis *from Gansu, Yunnan, Sichuan, Jiling, Shanxi, Hunan, and Hubei were collected by ourselves; *A. gigas* from Republic of Korea (ROK) and Democratic People's Republic of Korea (DPRK) were collected by Dr. H. Xiu of National Products Chemistry Laboratory, Department of Applied Biological and Environmental Chemistry, Seoul National University. The plant materials were collected in September to October after they had been cultivated for 2 years. Three-year-old* A. membranaceus* var. *mongholicus *roots were collected from Shanxi Province [17]. The authentication of all plant materials was identified morphologically by Dr. Tina Dong at Hong Kong University of Science and Technology. The voucher specimens were deposited in the Centre for Chinese Medicine R&D at the Hong Kong University of Science and Technology.

 Ferulic acid was from Sigma (St. Louis, MO); paeonol was purchased from National Institute for the Control of Pharmaceutical and Biological Products (Beijing, China); butylphthalide, and Z-butylidenephthalide, and senkyunolide A were purchased from Weikeqi Biotechnology Co. (Sichuan, China); Z-ligustilide was kindly provided by Prof. Pengfei Tu, Medical College of Peking University. All chemical standards were proven to have purity of 98% or above basing on their GC profile and MS data. Analytical- and HPLC-grade reagents were from Merck (Darmstadt, Germany). 

### 2.2. Extraction of *Angelica* Roots and DBT

In the herbal extraction, 50 mL of an ethyl acetate solution was added onto 0.5 g ground powders of *Angelica* roots. Ethyl acetate was used as to maximize the extraction. The mixture was then sonicated at ultrasonic bath (240 W) for 30 min. The supernatant after centrifugation (2,500 × g at 4°C for 10 min) was collected for GC/MS/MS analysis. In water extraction of *Angelica* root, about 15 g of root was weighed, boiled in 120 mL of water for 2 hours, and extracted twice. For the second extraction of *Angelica* roots, the residue from the first extraction was filtered and the same extracting conditions were applied. The extract was dried under vacuum and stored at −80°C, which was used for rapid resolution liquid chromatography (RRLC) analysis and biological determination.

 Thirty gram of sliced *Angelica* roots from Gansu, China, Republic of Korea (ROK), and Democratic People's Republic of Korea (DPRK) were weighed separately and mixed with AR separately to form three different DBT: DBT with *A. sinensis *from Gansu, China (denoted as DBT-China), DBT with *A. gigas* from ROK (denoted as DBT-ROK), and DBT with *A. gigas *from DPRK (denoted as DBT-DPRK). The mixture was boiled in 8 volumes of water (v/w) for 2 hours and extracted twice; this extraction followed the ancient recipe that had been shown to have the best extracting condition [11, 18, 19]. The extract was dried under vacuum and stored at −80°C. 

### 2.3. RRLC-QQQ-MS/MS

The water extract of DBT were quantitative analyzed by RRLC-QQQ-MS/MS. An Agilent 1200 series system (Agilent, Waldbronn, Germany) equipped with a degasser, a binary pump, an autosampler, and a thermo-stated column compartment was used for the analysis. The chromatographic condition was as described previously [20]. For the MS/MS analysis, an Agilent QQQ-MS/MS (6410A) equipped with an ESI ion source was used for all analyses. The marker chemicals including calycosin-7-O-**β**-D-glucoside, ononin, calycosin, astragaloside IV, and formononetin in DBT were quantified as described previously [20]. 

### 2.4. GC-QQQ-MS/MS Analysis

#### 2.4.1. GC-QQQ-MS/MS

 For the quantitative analysis of *Angelica* roots' extract, Agilent 7000 GC/MS/MS series system (Agilent, Waldbronn, Germany) was applied, which was equipped with an Agilent 7890A gas chromatography and GC-QQQ MassHunter workstation software. The extract was separated on an Agilent HP-5MS capillary column (250 *μ*m × 30 m × 0.25 *μ*m). The column initial temperature was kept at 70°C, and then the temperature was increased to 280°C at a rate of 10°C/min. Pulsed splitless injection was conducted, injecting 1 *μ*L of sample. The carrier gas was helium, at a flow rate of 1.2 mL/min. The spectrometer was operated in full-scan electron-impact (EI) mode, and the ionization energy was 70 eV. The inlet and ionization source temperature were 250°C and 280°C, respectively, and the solvent delay time was 3.5 min. The detector setting was gain, and tuning setting was EI high sensitivity autotune. For the MS/MS analysis, the suitable precursor ion and two product ions were chosen for acquisition in MRM mode for ferulic acid, butylphthalide, Z-butylidenephthalide, senkyunolide A, Z-ligustilide, and paeonol (internal standard). The fragmentor voltage and collision energy values were optimized to obtain the highest abundance. Agilent MassHunter software was used for data acquisition and processing. 

#### 2.4.2. Method Validation

To validate the analytic method, the linearity, sensitivity, precision, repeatability, and accuracy of the analytes were determined. For the linearity, the calibration curve of each chemical was constructed using a range of concentrations of working standards, and each line was based on six different concentrations. The limit of detection (LOD) and limit of quantification (LOQ) were used to evaluate the sensitivity. The LOD was estimated with a signal 3 times higher than that of the baseline noise, while the LOQ was 10 times higher. The precision was determined by intraday and interday variations, which were performed by analyzing standard solutions during a single day (*n* = 6) and on three executive days (*n* = 6), respectively. For repeatability test, five independent sample solutions were prepared in the procedures of sample preparation. The accuracy was evaluated as the percentage recovery of analytes in the spiked samples. The recoveries were calculated by the following formula: recovery (%) = 100 × (amount found − original amount)/amount spiked. RSD was used to describe precision, repeatability, and recovery.

### 2.5. Cell Proliferation and Differentiation Assay

Human mammary epithelial carcinoma MCF-7 cells, human osteosarcoma MG-63 cells, and human embryonic kidney fibroblast (HEK) 293T cells were obtained from American Type Culture Collection (ATCC, Manassas, VA). MCF-7 cells and MG-63 cells were grown in modified Eagle's medium (MEM), supplemented with 10% fetal bovine serum, 2 mM L-glutamine, 0.1 mM nonessential amino acids, 1 mM pyruvate, 100 units/mL penicillin, and 100 units/mL streptomycin in a humidified CO_2 _(5%) incubator at 37°C. HEK-293T cells were cultured in Dulbecco's modified Eagle's medium (DMEM), supplemented with 10% fetal bovine serum, 100 units/mL penicillin, and 100 units/mL streptomycin in a humidified CO_2_ (5%) incubator at 37°C. All culture reagents were purchased from Invitrogen Technologies (Carlsbad, CA). The cell proliferation was measured by 3-(4,5-dimethylthioazol-2-yl)-2,5-diphenyltetrazolium bromide (MTT) assay. In brief, cells were cultured in 96-well plates. After drug treatments for 48 hours, MTT (Sigma) was added onto the cultures and then extracted by DMSO solvent. The absorbance at 570 nm was measured. In parallel, the cell number was counted by a hemacytometer, as another cell proliferation parameter. 

### 2.6. Estrogen Promoter Assay in MCF-7 Cells

Three repeats of estrogen responsive elements (ERE: 5′-GGT CAC AGT GAC C-3′) were synthesized as described previously [21] and then subcloned into a luciferase-reporter vector called pTAL-Luc (BD Biosciences Clontech, San Jose, CA) to form pERE-Luc. This reporter was stably transfected to MCF-7 cells to generate stable cells according to a previous report [22]. To determine the estrogenic properties, different herbal extracts were applied onto the cultures for 48 hours. Afterward, the medium was aspirated, and MCF-7 cells were washed by ice-cold PBS. The cell lysate was centrifugation at 16,000 × g and 4°C for 10 min, and the supernatant was collected. Fifty *μ*L of the supernatant was used to perform the luciferase assay (Tropix Inc., Bedford, MA), and the activity was normalized by equal amount of protein.

### 2.7. Osteogenic Assay in MG-63 Cells

The differentiation of MG-63 cells was determined by the expression of alkaline phosphatase (ALP), an indicative biomarker for later stage of osteoblast differentiation. ALP activity in MG-63 cells was measured by the hydrolysis of *p*-nitrophenyl phosphate (Sigma) as described previously [23, 24]. Briefly, the cells were plated in 33-mm dishes and incubated with phenol red-free medium plus 10% charcoal-stripped fetal bovine serum. After 48 hours, cells were washed with cold phosphate-buffered saline (PBS; pH 7.4), scraped into 0.2 mL of 0.1% Triton X-100, and rapidly frozen and thawed three times to complete the lysis. The homogenate (100 *μ*L) was added to the substrate containing 10 mM *p*-nitrophenyl phosphate in 100 mM diethanolamine buffer (pH 10.5) supplemented with 0.5 mM MgCl_2_. After 30 min of incubation at 37°C, the reaction was terminated by the addition of 0.1 mL of 2 M NaOH, and the alkaline phosphatase activity was determined spectrophotometrically (405 nm) by measuring *p*-nitrophenyl released from the substrate. The enzyme activity was expressed as *μ*mol of substrate cleaved per mg of cell protein.

### 2.8. EPO Assay in Cultured HEK293T Cells

For the analyses of EPO expression in HEK293T cells, the cultures were treated with different extracts. Total RNA was isolated by TRIzol reagent and reverse transcribed into cDNAs according to the manufacturer's instructions (Invitrogen). Real-time PCR was performed by using SYBR Green Master mix and ROX reference dye according to the manufacturer's instructions (Applied Bioscience, Foster City, CA). The primers were 5′-ACT TTC CGC AAA CTC TTC CG-3′ and 5′-TGA ATG CTT CCT GCT CTG GG-3′ for human EPO (330 bp; NM_000799.2). The 18S rRNA was used as an internal control in all cases, and its primer sequences were 5′-TGT GAT GCC CTT AGA TGT CC-3′ and 5′-GAT AGT CAA GTT CGA CCG TC-3′ (320 bp; NR_003286). SYBR green signal was detected by M×3000 ptm multiplex quantitative PCR machine (Stratagene, La Jolla, CA). Transcript levels were quantified by using ΔΔCt value method [25], where the values of target genes were normalized by the18S rRNA in the same sample at first before comparison. PCR products were analyzed by gel electrophoresis and melting curve analysis to confirm the specific amplification.

### 2.9. Statistical Analysis and Other Assays

 Protein concentrations were measured routinely by Bradford's method with a kit from Bio-Rad Laboratories (Hercules, CA). Statistical tests were done by using one-way analysis of variance. Data were expressed as mean ± SD, where *n* = 4. Statistically significant changes were classified as significant (*) where *P* < 0.05 and highly significant (**) where *P* < 0.01.

## 3. Results

### 3.1. Chemical Composition of Different *Angelica* Root

**Figure 1:**
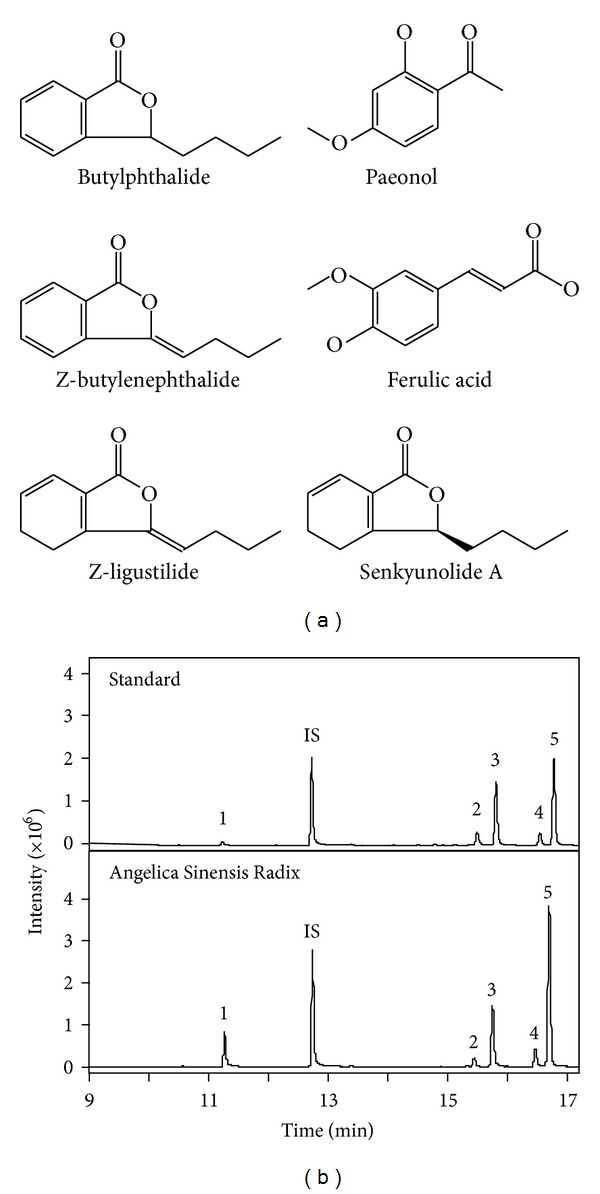
GC-MS/MS MRM chromatograms of extracts from *Angelica* roots. (a) Chemical structures of ferulic acid, butylphthalide, Z-butylidenephthalide, senkyunolide A, Z-ligustilide, and paeonol were shown. (b) GC-MS MRM chromatograms of ethyl acetate extracts of *Angelica* roots (from Gansu, China). One *μ*L was injected. The information for each peak was indicated: (1) ferulic acid, (2) butylphthalide, (3) Z-butylidenephthalide, (4) senkyunolide A, (5) Z-ligustilide, and (IS) paeonol. Representative chromatograms are shown, *n* = 3.

 The amounts of ferulic acid, butylphthalide, Z-butylidenephthalide, senkyunolide A, and Z-ligustilide in the extracts deriving from *Angelica* roots were determined as marker chemicals. These chemicals were considered to possess known biological functions of *Angelica* root, as described previously [26]. The chemical structure of these markers (Figure 1(a)) and their characteristics in the MS/MS (see Table 1 in Supplementary Material at http://dx.doi.org/10.1155/2013/483286) were revealed. In MS/MS analysis, each calibration curve was obtained from six different concentrations of marker chemicals. The squared values of all correlation coefficients (*r* ^2^) of these calibration curves were higher than 0.990 in MS/MS analysis. The LOD and LOQ were determined at a S/N of 3 and 10, respectively (Supplementary Table 2). The precision, repeatability, and recovery were determined (Supplementary Table 3). The results showed that the GC-QQQ-MS/MS method was precise, accurate, and sensitive enough for simultaneous, quantitative evaluation of marker chemicals in the extracts of *Angelica* roots. 

**Figure 2:**
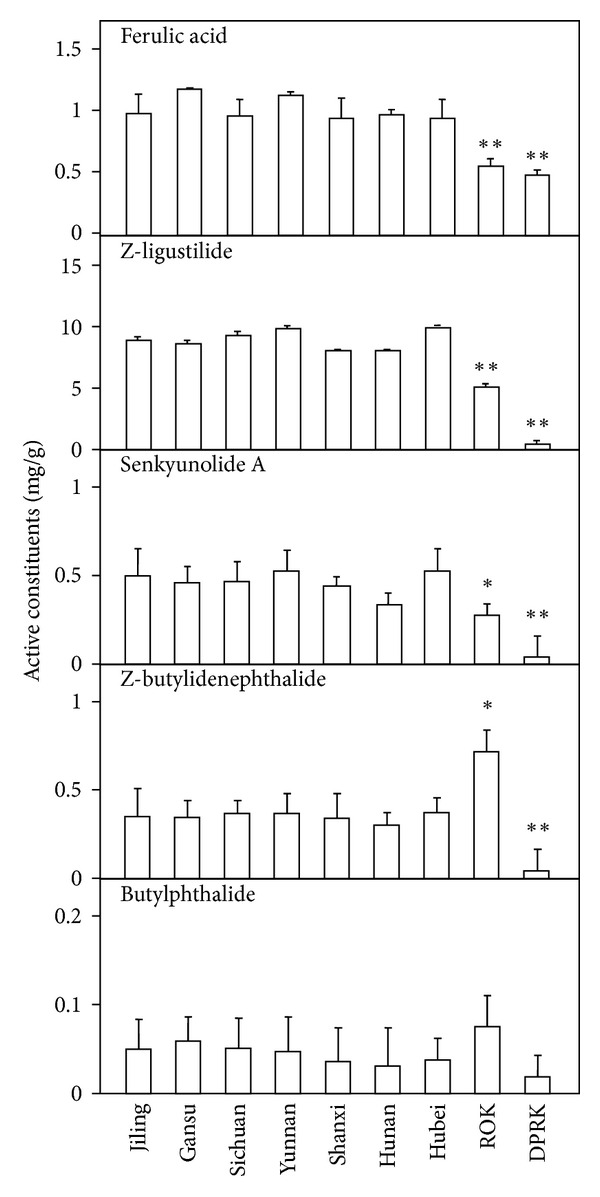
Comparison of five chemical markers in the extracts of *Angelica* roots. The ethyl acetate extracts of *Angelica* roots was analyzed by GC-QQQ-MS/MS. The detailed protocol was described in Material and Methods. Values are expressed in milligrams per gram of dry material and in mean ± SD, where *n* = 3. Statistical comparison was made with *Angelica* roots from Gansu, China, *P < 0.05; **P < 0.01.

**Figure 3:**
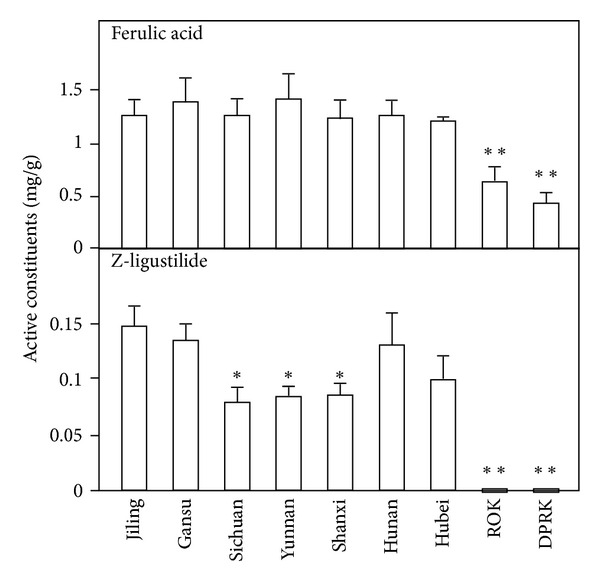
Amount of ferulic acid and Z-ligustilide in water extracts of *Angelica* roots. The water extracts of *Angelica* roots was analyzed by  RRLC-QQQ-MS/MS. Values are expressed in milligrams per gram of dry material and in mean ± SD, where *n* = 3. Statistical comparison was made with *Angelica* roots from Gansu, China, *P < 0.05; **P < 0.01.

 Typical MS/MS chromatograms of the mixed standards, or in *Angelica* extracts, were shown in Figure 1(b), and the amounts of different marker chemicals were quantified according to their MS patterns. The *Angelica* roots from China, extracted by ethyl acetate, showed great similarity chemically, which contained higher amounts of ferulic acid, senkyunolide A, and Z-ligustilide than that from Korea (Figure 2). The amounts of butylphthalide and Z-butylidenephthalide were relatively lower in these samples. In contrary, *Angelica* roots from ROK showed higher amount of butylphthalide and Z-butylidenephthalide, while all tested chemicals were found to be lower in *Angelica* roots from DPRK. Water is common solvent in preparing *Angelica* root decoction. Therefore, the water extract of nine *Angelica* roots were also quantified by measuring the amount of ferulic acid and ligustilide. The amount of ferulic acid was almost the same as that in the extract of ethyl acetate (Figure 3). However, the amount of ligustilide was dramatically decreased after boiling, which could be a possible reason of the loss of volatilized ligustilide during the heating. 

 In the common usage, *Angelica* root is prepared together with other herbs in boiling water and resulted in a herbal decoction, that is a herbal formula. Amongst various herbal formulae, DBT is a well-studied Chinese herbal decoction [13–16] which contains AR and *Angelica* root in 5 : 1 ratio. The key chemicals including ferulic acid, calycosin-7-O-**β**-D-glucoside, ononin, calycosin, astragaloside IV, and formononetin in DBT, prepared from different sources of *Angelica* root, were determined and compared (Table 1). The amount of ferulic acid was higher in DBT-China than that in DBT-ROK and DBT-DPRK, which could be an outcome of higher ferulic acid contained within China *Angelica* root. Interesting, AR-derived chemicals including calycosin, astragaloside IV, and formononetin were markedly higher in DBT-China than the others (Table 1): this was the coboiling effect of different *Angelica* roots on the solubility of AR-derived chemicals. In general, the compatibility amongst all the herbs within a decoction is an important principle of TCM theory, which ensures the high amounts of active ingredients and full pharmacological properties of TCM decoctions.

### 3.2. Biological Properties of *Angelica* Roots and DBT

#### 3.2.1. The Osteogenic Effect of *Angelica* Roots and DBT

**Figure 4:**
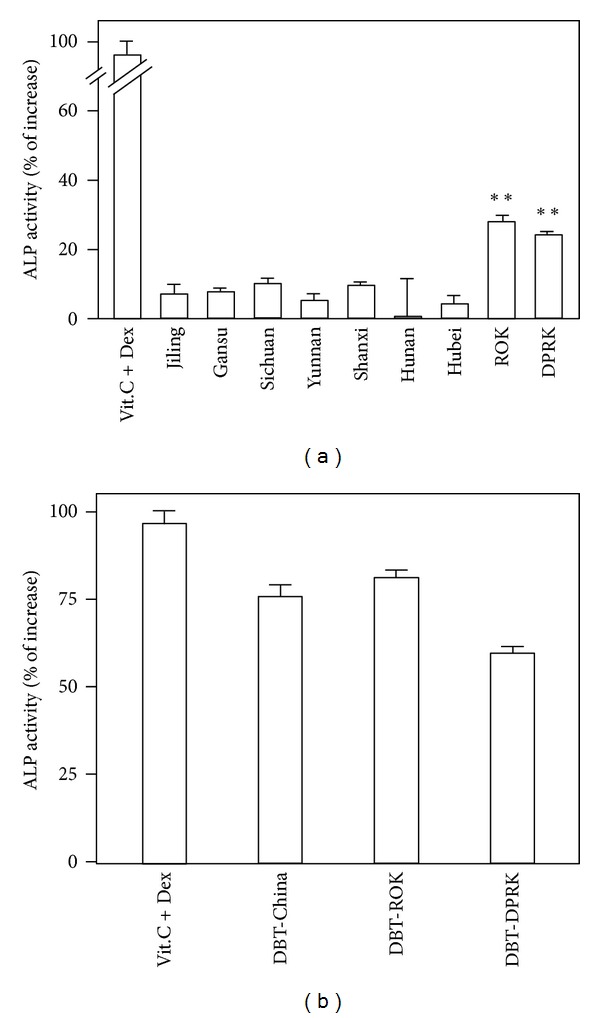
*Angelica* root extract increases the enzymatic activity of alkaline phosphatase in cultured MG-63 cells. (a) The water extracts of *Angelica* roots at 0.5 mg/mL were applied onto cultured MG-63 cells for 48 hours to determine the enzymatic activity of alkaline phosphatase (ALP). Dexamethasone (50 nM) together with vitamin C (250 *μ*M) was used as a control in MG-63 cells. Values are expressed in percentage of increase as compared with control cultures (without herbal extract) and are in mean ± SD, where *n* = 4, each with triplicate samples. **P < 0.01. (b) Cultures were treated as in (a) with different DBT at 0.7 mg/mL to determine the enzymatic activity of ALP. Values are expressed in percentage of increase as compared with control cultures (without herbal extract) and are in mean ± SD, where *n* = 4, each with triplicate samples.

The biological properties of water extracts from different *Angelica* roots were compared here, including osteogenic, estrogenic, and hematopoietic effects. The water extracts were chemical standardized as shown in Figure 3. MG-63 cell is a common cell line used in analyzing osteoblast differentiation [23, 24]. The level of ALP, a differentiation marker of osteoblast, expressed by cultured MG-63 cells, was determined here. The results showed that the level of ALP was only increased by applied *Angelica* extracts from ROK and DPRK (Figure 4(a)); however, the application of *Angelica* root extracts from China did not show a robust increase. The osteogenic effects of different DBT, prepared from different sources of *Angelica* roots, were also compared (Figure 4(b)). The expression levels of ALP induced by the three DBT preparations were rather similar.

#### 3.2.2. The Estrogenic Effect of *Angelica* Roots and DBT

**Figure 5:**
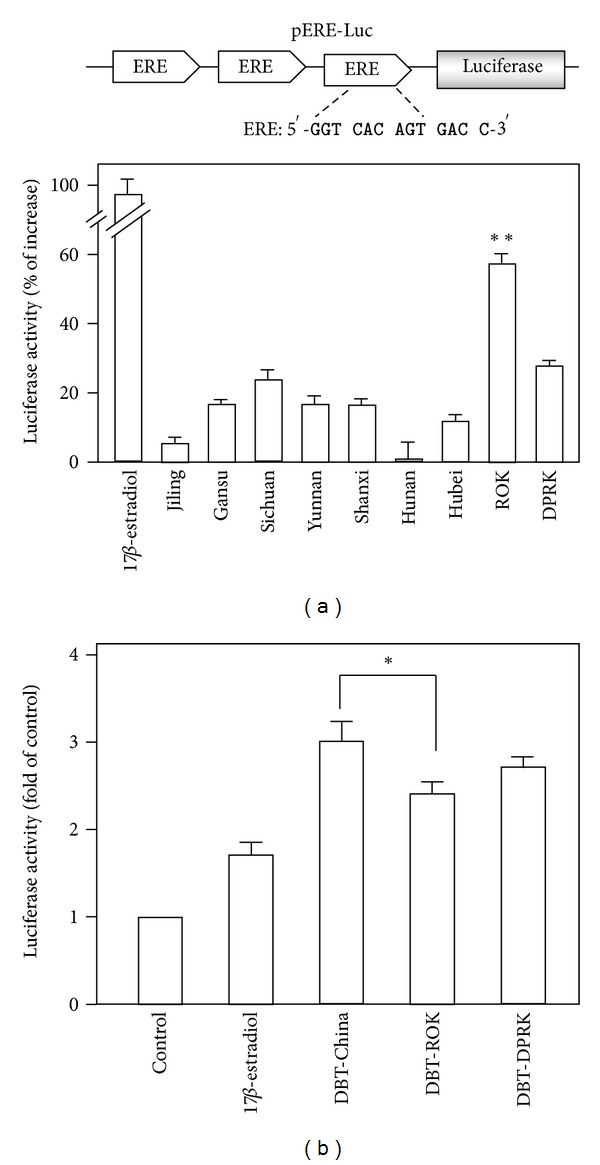
*Angelica* root extract and DBT stimulate transcriptional activity of estrogen-responsive element of MCF-7 cells. (a) Three repeats of estrogen responsive elements (ERE: 5′-GGT CAC AGT GAC C-3′) were subcloned into a luciferase-reporter vector called pERE-Luc (upper panel). This reporter was stably transfected to MCF-7 cells, which were treated with *Angelica* root extracts at 0.5 mg/mL for 48 hours. 17-**β**-estradiol (100 nM) was used as a positive control. Values are expressed in percentage of increase as compared with control cultures (without herbal extract) and are in mean ± SD, where *n* = 4, each with triplicate samples. **P < 0.01. (b) Cultures were treated as in (a) with different DBT at 0.7 mg/mL. Values are expressed in the fold of change as compared with control cultures (without herbal extract) and are in mean ± SD, where *n* = 4, each with triplicate samples. *P < 0.05.

To investigate the estrogenic activity, MCF-7 cells stably transfected with pERE-Luc were employed. The water extracts of different *Angelica *roots were applied onto the cultures for 48 hours. The promoter (ERE-) driven luciferase activity was subsequently determined. The expression of luciferase, carried in pERE-Luc, was markedly induced by *Angelica* roots from ROK (~60% increase), while the other *Angelica* extracts deriving from China showed only about 20% of the increase (Figure 5(a)). However, when DBT was produced with different *Angelica* roots, the DBT prepared from Chinese *Angelica* roots (DBT-China) showed the strongest activity in inducing the luciferase activity (Figure 5(b)), which is slightly higher than DBT-DPRK. Interestingly, the DBT-ROK showed the lowest activity. A positive control, 17-**β**-estradiol at 100 nM, caused ~2-fold increase in the promoter activity. 

#### 3.2.3. The Erythropoietic Effect of DBT

**Figure 6:**
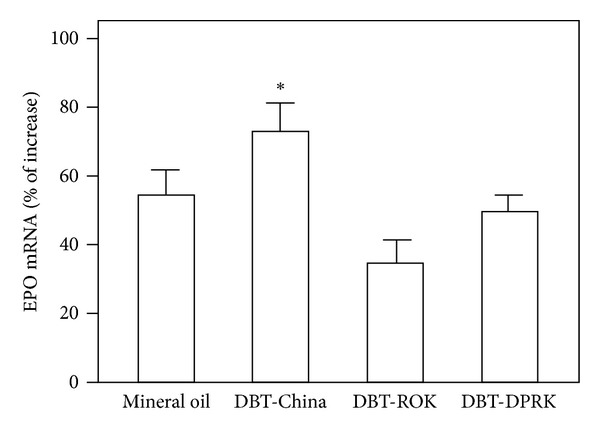
DBT induces EPO expression in cultured HEK293T cells. Cultured HEK293T cells were treated with 0.7 mg/mL of different DBT for 48 hours. The level of EPO mRNA was revealed by real-time PCR, while 18S rRNA was used as an internal control for normalization. The overlayering of mineral oil served as a positive control. Values are expressed as the percentage of increase to basal reading (untreated culture) and are in mean ± SD, where *n* = 4, each with triplicate samples. *P < 0.05.

 Human embryonic kidney fibroblast cell line, HEK293T, has been shown to be an excellent *in vitro* model in studying the physiological regulation of EPO expression [27]. To reveal the tropic effect of the three DBT, the herbal water extracts were applied in cultured HEK293T cells for 2 days. After the treatment, total RNA was collected from the treated cells and subjected to real-time quantitative PCR analysis by using specific primers flanking EPO mRNA. Mineral oil, layering on the top of cultures (to block gaseous exchange for mimicking hypoxia) as a positive control for EPO induction, increased the level of EPO mRNA by ~60% (Figure 6). The application of DBT-China induced the amount of EPO mRNA by ~80%. The extract of DBT-ROK also increased the EPO mRNA by ~45%, but the response was significantly less than that of DBT-China. Similar case was observed in DBT-DPRK treatment. 

### 3.3. Cytotoxicity of *Angelica* Root Extracts and DBT

**Figure 7:**
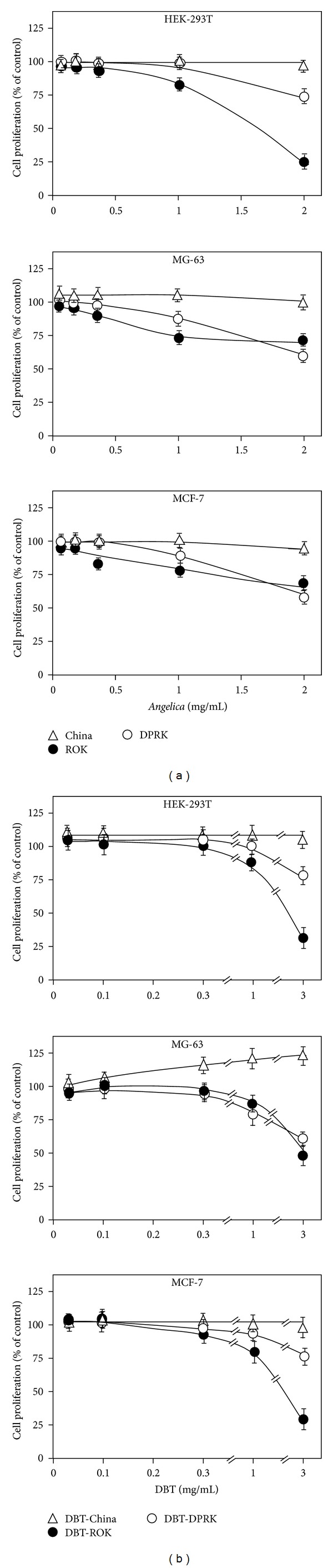
Effect of *Angelica* root extracts and DBT on cell growth. (a) Cultured HEK-293T cells, MG-63 cells and MCF-7 cells were treated with various concentrations of *Angelica* roots for 48 hours, and the cell viability was determined by MTT assay. (b) Cultures were treated as in (a) with various concentrations of DBT to determine the cell proliferation by MTT assay. Values are expressed in percentage of change as compared with control cultures (without herbal extract), and are in mean ± SD, where *n* = 4, each with triplicate samples.

We first determined the cell viability of the water extracts of *Angelica* roots from Gansu of China, ROK, and DPRK in cultured HEK-293 cells, MG-63, cells and MCF-7 cells. Cells were treated with 0.03 to 2 mg/mL *Angelica* root extracts for 48 hours. As shown in Figure 7(a), the treatment with *Angelica* root extracts from ROK and DPRK decreased the cell proliferation in a dose-dependent manner, in particular under the high concentration being used here. Cultured HEK-293 cells were found to be more sensitive to *Angelica* roots from ROK as compared to other two cells lines, which caused cell death to almost 80%. On the other hand, high concentration of *Angelica* root extract from Gansu, China showed no obvious cell toxicity to the cell lines. 

 The DBT made with *Angelica* roots from ROK and DPRK also decreased the cell proliferation in a dose-dependent manner (Figure 7(b)). The cell viability of HEK-293 and MG-63 was greatly inhibited by DBT-ROK, and less than 25% of the cells were survived after the drug treatment. The DBT-China again showed no effect on the cell viability on HEK-293 cell and MCF-7 cell. In contrast, the cell proliferation of MG-63 cell was increased after the application of DBT-China decoction. Indeed, the cell proliferation was also a biomarker of osteogenic effect for this cell line.

## 4. Discussion

 According to TCM theory, the quality of Chinese medicines produced in different areas could be different. The authentic source, or “Daodi” (in Chinese), is a general guidance for the proper usage of Chinese herbs. Our study here indicated that *Angelica* roots from different regions of China showed similarity both in chemical composition and in biological functions, while large difference was observed among *Angelica* roots from Korea, both ROK and DPRK. Historically, Gansu province is known to produce the best *Angelica* root in China; our previous studies also indicated that high amounts of ferulic acid and Z-ligustilide were found in the *Angelica* roots from Gansu [28], as compared with those from other places in China. However, the current result showed that *Angelica* roots from other places in China also contained high amount of the chemical markers. The development of standardized planting and good agricultural practice (GAP) is being used now, and which could be a main reason for this better herb from different parts of China. The chemical difference between *Angelica* roots from China and Korea could be a result of that they belonged to different species, while the difference between *A. gigas* from ROK and DPRK might be due to the cultivation in different places. When using the TCM, therefore, the species and the production areas of herbs should be considered.

The cause of cell toxicity of *Angelica* roots from Korea in cultured cells is not known; however, the Korea *Angelica* roots are known to contain distinct chemicals. Studies showed that the *A. gigas* contains pyranocoumarin compounds as major active principles, including decursin [29], its isomer decursinol angelate [30], and decursinol [31]. Decursin was reported to inhibit the growth and survival of metastatic prostatic cancer cells [32, 33], while both decursin and decursinol angelate had an effect on antiproliferation by inducing apoptosis in cervical cancer cells. Moreover, decursin was also cytotoxic against normal cells [34]. Our study also showed that *A. gigas* roots had antiproliferation effect on the three tested cell lines, while all the *Angelica* roots from China had no such effect. *Angelica* roots from ROK and DPRK could be used as potential anticancer drug, while *Angelica* roots from China may have better effect in stimulating blood circulation, since it has high amount of ferulic acid and Z-ligustilide, and these two chemicals could decrease the ADP-induced platelet aggregation [26, 35]. 

 Herbal formula is the major form of clinical application of TCM. Indeed, the traditional therapeutic formulations consist of a combination of several herbs, however, which is not simply the sum of the single herbs, and complex interactions of multiple herbs are thought to maximize therapeutic efficacy and to prevent potential adverse effects [18]. For example, our previous study showed that the function of DBT is better than the single herb of ASR, AR, and the mixture of AR and ASR without boiling [18]. Our study here showed that the *Angelica* roots from ROK showed better effect in inducing the ALP activity and the ERE driven luciferase activity, but DBT-ROK did not show any superiority than other DBT. In contrast, DBT-China showed better effect on all the bioactivities tested here, which could due to the higher amount of flavonoids contained within DBT-China, as our previous study showed that the flavonoids from AR were essential for the biological functions of DBT [24, 27, 36]. However, more studies are needed to account for the better biological effects of DBT-China. Our results suggest that it is more reasonable to use *A. sinensis* to prepare DBT, since the major function of DBT is to nourish the “Qi” and enrich the “Blood,” so the cytotoxic effect produced by the *A. gigas* to the cancer cells and normal cells indicates that it is less suitable than *A. sinensis* to be the ingredient in DBT. 

## 5. Conclusion

 In conclusion, our study indicates that the species and source of herb should be considered during the preparation of herbal formulae; this optimization achieve their maximum biological efficacy and minimal toxicity. 

## Supplementary Material

Supplementary Table 1: Mass spectra properties of marker chemicals. For the MS/MS analysis, the suitable precursor ion and two product ions were chosen for acquisition in MRM mode for ferulic acid, butylphthalide, Z-butylidenephthalide, senkyunolide A, Z-ligustilide, and paeonol (internal standard). The fragmentor voltage and collision energy values were optimized to obtain the highest abundance. Agilent MassHunter so/ware was used for data acquisition and processing.
Supplementary Table 2: Calibration curves, LOD and LOQ of five markers. For the linearity, the calibration curve of each chemical was constructed using a range of concentrations of working standards, and each line was based on six different concentrations. The limit of detection (LOD) and limit of quantication (LOQ) were used to evaluate the sensitivity. The LOD was estimated with a signal 3 times higher than that of the baseline noise, while the LOQ was 10 times higher.
Supplementary Table 3: Precision, repeatability and recovery of markers. The precision was determined by intraday and interday variations, which were performed by analyzing standard solutions during a single day (*n* = 6) and on three executive days (*n* = 6), respectively. For repeatability test, five independent sample solutions were prepared in the procedures of sample preparation. The accuracy was evaluated as the percentage recovery of analytes in the spiked samples. The recoveries were calculated by the following formula: recovery (%) = 100 × (amount found – original amount)/amount spiked. RSD was used to describe precision, repeatability, and recovery.

## Figures and Tables

**Table 1:** Quantitative assessment of six marker chemicals in DBT with different *Angelica* roots.

Chemical	Amount^ a^		
	DBT-China	DBT-ROK	DBT-DPRK
Ferulic acid	1438.15 ± 92.73**	418.42 ± 22.47	321.39 ± 14.17
Calycosin-7-O-*β*-D-glucoside	236.42 ± 30.72	253.72 ± 27.49	241.53 ± 33.08
Ononin	66.23 ± 4.08	84.72 ± 10.52	73.44 ± 8.46
Calycosin	219.98 ± 24.77**	166.06 ± 19.68	173.63 ± 10.06
Astragaloside IV	354.73 ± 36.26*	268.46 ± 30.15	309.23 ± 27.42
Formononetin	157.52 ± 18.72**	98.47 ± 16.49	81.25 ± 21.08

^a^Values are expressed in *μ*g/g dried extracts of DBT, mean ± SD, where *n* = 3, each with triplicate samples.

*P < 0.05 as compared with two other DBT.

**P < 0.01 as compared with two other DBT.

## Abbreviations

DBT: Danggui Buxue Tang
TCM: Traditional Chinese medicine.
