# Supplementary material for: Chemical and Biological Assessment of Angelica Roots from Different Cultivated Regions in a Chinese Herbal Decoction Danggui Buxue Tang
Source: Evid Based Complement Alternat Med. 2013 Feb 5;2013:483286. doi: 10.1155/2013/483286 (PMC3576791; doi:10.1155/2013/483286)
Supplement: Supplementary file 1 — Supplementary Table 1: Mass spectra properties of marker chemicals. For the MS/MS analysis, the suitable precursor ion and two product ions were chosen for acquisition in MRM mode for ferulic acid, butylphthalide, Z-butylidenephthalide, senkyunolide A, Z-ligustilide, and paeonol (internal standard). The fragmentor voltage and collision energy values were optimized to obtain the highest abundance. Agilent MassHunter so/ware was used for data acquisition and processing. Supplementary Table 2: Calibration curves, LOD and LOQ of five markers. For the linearity, the calibration curve of each chemical was constructed using a range of concentrations of working standards, and each line was based on six different concentrations. The limit of detection (LOD) and limit of quantication (LOQ) were used to evaluate the sensitivity. The LOD was estimated with a signal 3 times higher than that of the baseline noise, while the LOQ was 10 times higher. Supplementary Table 3: Precision, repeatability and recovery of markers. The precision was determined by intraday and interday variations, which were performed by analyzing standard solutions during a single day (n = 6) and on three executive days (n = 6), respectively. For repeatability test, five independent sample solutions were prepared in the procedures of sample preparation. The accuracy was evaluated as the percentage recovery of analytes in the spiked samples. The recoveries were calculated by the following formula: recovery (%) = 100 × (amount found – original amount)/amount spiked. RSD was used to describe precision, repeatability, and recovery. [file 483286.f1.pdf]

Supplementary Table 1. Mass spectra properties of marker chemicals.

Chemical	Formula	Calculated mass [M]	Precursor ion [a]	Collision energy [b]	Production ion [c]	Retention time [d]
**Ferulic acid**	$C_{10}H_{10}O_4$	194.10	149.6	10<br>20	135<br>107	11.251
**Paeonol**	$C_9H_{10}O_3$	166.17	150.6	10<br>40	95<br>52	12.757
**Butylphthalide**	$C_{12}H_{14}O_2$	190.23	132.6	20<br>40	77<br>51	15.527
**Z-Butylidenephthalide**	$C_{12}H_{12}O_2$	188.22	158.6	10<br>20	131<br>103	15.852
**Senkyunolide A**	$C_{12}H_{16}O_2$	192.25	106.7	10<br>10	79<br>77	16.580
**Z-Ligustilide**	$C_{12}H_{14}O_2$	190.10	147.7	20<br>40	105<br>77	16.823

[a] The ion of relative highest intensity was used as the precursor ion for the quantification.

[b] The collision energy was optimized to have the greatest product ion intensity, which was the key factor in the MRM mode.

[c] Two product ions were used for the MRM analysis. The upper one was used for quantitative analysis and the lower one was for qualitative analysis, which could guarantee the precision of analytes.

[d] The retention time was determined by 3 different individual analyses (n = 3).

Supplementary Table 2. Calibration curves, LOD and LOQ of five markers.

Chemical	Calibration curve[a]	Correlation coefficient (r^2)	Linear range	LOD [b] (ng/ml)	LOQ[c] (ng/ml)
**Z-Ligustilide**	y=0.7169x-0.0497	0.9945	0.1-50	7.08	33.3
**Ferulic acid**	y=0.7984x-0.2864	0.9903	0.1-50	2.77	12.5
**Butylphthalide**	y=14.0273x-0.1603	0.9970	0.02-5	0.32	1.3
**Z-Butylidenephthalide**	y=4.4408x-0.0611	0.9955	0.02-5	0.47	2
**Senkyunolide A**	y=2.3792x-0.0417	0.9922	0.02-5	2.71	13

[a]The calibration curves were constructed by plotting the peak areas versus the concentration of each analyte. Each calibration curve included six data points.

[b]LOD refers to the limits of detection.

[c]LOQ refers to the limits of quantification.

Supplementary Table 3. Precision, repeatability and recovery of markers.

Chemical	Precision				Repeatability (*n*=5)		Recovery[a] (*n*=5)	
	Intra-day[b] (*n*=6)		Inter-day[c] (*n*=6)					
	Mean (ng/mL)	RSD (%)	Mean (ng/mL)	RSD (%)	Mean (ng/mL)	RSD (%)	Mean (%)	RSD (%)
**Z-Ligustilide**	0.98	1.98	0.91	3.29	17.67	2.98	96.91	3.31
**Ferulic acid**	1.04	0.26	1.44	1.58	1.41	1.19	97.57	1.85
**Butylphthalide**	1.01	1.48	0.93	1.39	0.91	1.73	99.90	1.66
**Z-Butylidenephthalide**	1.07	2.76	1.02	2.36	0.10	2.03	100.30	2.10
**Senkyunolide A**	0.96	3.01	1.04	4.05	0.89	2.61	98.75	2.21

[a] Recovery (%) =100 × (amount found－original amount)/amount spiked. The data were presented as average of three independent determinations, and the SD was ＜5% of the mean, which was not shown for clarity.

[b] The intra-day analysis refers to the sample examined for six replicates within one day.

[c] The inter-day analysis refers to the sample examined in duplicates over three consecutive days.
